# The Role of the Gastric Hormones Ghrelin and Nesfatin-1 in Reproduction

**DOI:** 10.3390/ijms222011059

**Published:** 2021-10-14

**Authors:** Martha A. Schalla, Andreas Stengel

**Affiliations:** 1 Charité Center for Internal Medicine and Dermatology, Department for Psychosomatic Medicine, Berlin Institute of Health, Charité-Universitätsmedizin Berlin, Corporate Member of Freie Universität Berlin, Humboldt-Universität zu Berlin, 12203 Berlin, Germany; martha.schalla@charite.de; 2 Department of Psychosomatic Medicine and Psychotherapy, Medical University Hospital Tübingen, 72076 Tübingen, Germany

**Keywords:** gastric, ghrelin, hormone, HPG axis, nesfatin-1, peptide, reproduction

## Abstract

Ghrelin and nesfatin-1 are enteroendocrine peptide hormones expressed in rat X/A-like and human P/D1cells of the gastric mucosa. Besides their effect on food intake, both peptides are also implicated in various other physiological systems. One of these is the reproductive system. This present review illustrates the distribution of ghrelin and nesfatin-1 along the hypothalamus–pituitary–gonadal (HPG) axis, their modulation by reproductive hormones, and effects on reproductive functions as well as highlighting gaps in current knowledge to foster further research.

## 1. Introduction

In 1999, the long sought for endogenous ligand specific for the growth hormone secretagogue receptor (GHS-R) 1a, ghrelin, was identified [1]. This discovery was accompanied by the observation that ghrelin was expressed predominately in gastric tissue [1], which made ghrelin the only enteroendocrine hormone known to centrally stimulate food intake [2]. Over 20 years of research, however, showed that ghrelin, mostly in its active form after acylation by ghrelin-*O*-acyl transferase (GOAT), is implicated in many more functions than just the stimulation of growth hormone release and food intake; among others, it was shown to play a role in glucose and lipid metabolism, behavioral regulation, and cardiovascular functioning [3].

In the stomach, ghrelin is expressed in colocalization with nucleobindin-2 (NUCB2) [4], which consists of a 24-amino acid (aa) signal peptide followed by a 396-aa protein. Post-translational cleavage of the N-terminal of NUCB2 by prohormone convertase -1/3 also yields, besides two inactive peptides, nesfatin-2 and nesfatin-3, the active nesfatin-1 [1,4], a peptide hormone inhibiting food intake and body weight [5]. Noteworthy, antibodies commonly used to identify nesfatin-1 bind an epitope found in the peptide itself as well as in its precursor NUCB2 [1], thus when describing data based on usage of antibodies the term NUCB2/nesfatin-1 will be used. Although ghrelin and NUCB2/nesfatin-1 are stored in two separate populations of intracytoplasmic vesicles [4] they are located in the same gastric cells: in rat X/A-like and human P/D1cells [6]. In the last 15 years since its discovery, nesfatin-1 has been shown to have widespread effects and to be implicated in the metabolism of glucose and lipids, mediation of anxiety and depression, and exerts cardiovascular functions [7].

Thus, besides its food-intake- and body-weight-modulating effects [2,5], ghrelin and nesfatin-1 also exert pleiotropic effects. Since both peptides are expressed in organs of the hypothalamus–pituitary–gonadal (HPG) axis, are modulated by hormones implicated in reproduction and affect reproductive functions in various ways, this review aims to illustrate the current knowledge about these gastric peptides’ roles in reproduction. In order to foster further research, gaps in knowledge will be addressed as well.

## 2. Ghrelin

### 2.1. Expression in Tissues Implicated in Reproduction

#### 2.1.1. Hypothalamus

In the first report about ghrelin, it was observed that, although low in quantity, ghrelin is synthesized in the hypothalamus of the rat [1]. Subsequent examinations showed that ghrelin immunoreactive neurons were located in the internuclear space between the lateral hypothalamus (LH), arcuate (ARC), ventromedial (VMH), dorsomedial (DMH), and paraventricular hypothalamic nuclei (PVH) and the ependymal layer of the third ventricle in rats and mice [8]. Furthermore, ARC, LH and PVH were additionally innervated by ghrelin positive axons terminals and were found to bind biotinylated ghrelin, supposedly via the GHS-R [8].

Interestingly, ghrelin mRNA expression may be modulated by peripheral reproductive hormones, indicated by observations that hypothalamic preproghrelin mRNA expression was reduced 2.5 days after ingestion of estrogen- or testosterone-containing chow in goldfish [9]. Additionally, a study using semiquantitative RT-PCR showed alteration of hypothalamic expression of ghrelin mRNA with the estrous cycle [10], possibly due to changes in peripheral estradiol concentrations [11]; however, since this was not a quantitative measurement more investigations should follow.

Pregnancy also modulates ghrelin mRNA expression in the hypothalamus. However, there is data on reduced levels of ghrelin mRNA at day 15 of pregnancy in rats compared to non-pregnant controls [12], as well as reports about increased hypothalamic proghrelin levels throughout the whole pregnancy in rats compared to expression before pregnancy [13]. This difference in results could be due to different control conditions, however this hypothesis warrants further research.

#### 2.1.2. Pituitary

One of the tissues with the highest ghrelin mRNA levels is the pituitary [14,15]. Since its expression was observed also in the anterior part of this gland [15], an implication in reproduction of ghrelin is probable. This hypothesis is further corroborated by a reduction of pituitary ghrelin mRNA expression with the onset of puberty [16] and increased levels throughout pregnancy in rats [13]. Studies in the future should aim at identifying the specific factors responsible for the reduction of ghrelin expression in the pituitary as well as the underlying mechanism(s).

#### 2.1.3. Testis

Ghrelin was found to be expressed in the testis [14], with gene expression throughout the whole postnatal development [17]; however, its distribution is not completely clear. While some reports showed ghrelin immunoreactivity in interstitial Leydig cells and only minimally in Sertoli cells but not in germ cells [18], others found that after selective Leydig cell elimination no ghrelin protein was detected in the testicular interstitium [19]. More recently, ghrelin expression was also detected in early spermatocytes and round spermatids, in late spermatogonia, in spermatocytes up to early pachytenes, in spermatids, during the early maturation phase of spermiogenesis [20], as well as in head and tail of ejaculated and selected spermatozoa [21]. High ghrelin immunoreactivity was also reported in the efferent ductules, while a moderate intensity was seen in the epididymis, vas deferens and seminal vesicles, and low signal was detected in the rete testis [21]. Noteworthy, the enzyme responsible for activating ghrelin by acylation, GOAT, was also found to be expressed in human testis [22].

Interestingly, testicular ghrelin mRNA and protein expression were shown to be related to luteinizing hormone (LH), since they were almost undetectable after hypophysectomy and restored with application of a LH super-agonist [19]. In addition, ghrelin expression was increased due to estradiol supplementation in low-estrogen conditions in frogs [23] and negatively correlated to serum testosterone concentration [24]. Nevertheless, immune castration with recombinant GnRH inhibitor (GnRH-I) in boars did not alter testicular ghrelin expression [25]; thus, it needs to be further examined whether testicular ghrelin expression does indeed not depend on testosterone or if there are compensatory mechanisms under conditions of GnRH-I-induced hypogonadism ensuring ghrelin expression in the testis.

#### 2.1.4. Ovaries

Both ghrelin mRNA and peptide were observed to be expressed in the ovary [14,15]. The distribution of ghrelin expression in the ovary is not clear, due to partly contradicting data. Ghrelin was shown to be expressed in the ovarian interstitial cells [26], in the different forms of the corpus luteum [27,28] as well as in granulosa, theca and luteal cells [28], while ghrelin mRNA was also detected in the human tube and the ampulla in heifers [29]. In addition, GOAT, which is responsible for acylating and thus activating ghrelin, was also found to be expressed in human ovary [22].

The effect of estrous cycle on ghrelin expression was consistently reported. In prepubertal animals, ghrelin mRNA expression in ovarian follicles was reduced compared to cycling ones [30]. Additionally, inhibition of ovulation by a potent GnRH antagonist abolished cycle dependency of ghrelin mRNA expression [27]. Ghrelin and its mRNA seem to be expressed throughout the entire estrous cycle [27,31], however, ovarian mRNA expression of ghrelin was observed to be highest during diestrus and lowest during proestrus [10,27]. The expression of ghrelin in ovarian follicles [32] increased with follicle size, with highest expression in dominant or pre-ovulatory follicles [33], and correlated with estrogen levels in follicular fluid [30]. In the corpora lutea, ghrelin mRNA and protein expression was highest throughout its development [31,34], and reduced with corpora lutea regression [31]. To better understand the reasons for these changes in ovarian expression of ghrelin throughout the estrous cycle, further research should investigate the effect of specific reproductive hormone concentrations, especially estradiol and progesterone, on ovarian ghrelin mRNA and peptide. This would also help understand why during pregnancy, ghrelin mRNA was constantly expressed in rat ovary, but increased in the early phase and decreased in the late phase of pregnancy [27].

#### 2.1.5. Oocyte, Embryo, and Fetus

In sheep, ghrelin mRNA and peptide were found in oocytes, pre-implantation embryos [35], and in 12-day embryos in rats [16]. Similarly, in mice ghrelin was detected in the morula and advanced embryo stages [36]. Interestingly, while denuded oocytes [37] and metaphase II oocytes displayed the highest expression of mRNA and protein, the 2-cell stage showed high levels of expression of ghrelin mRNA and peptide while in the 4- and 8-cell stages and blastocysts the levels were low [35], indicating a decrease in expression with cell division.

Interestingly, in fetal blood and amniotic fluid a high level of desacyl ghrelin was detected [38], accompanied by a high rate of acyl ghrelin degradation in fetus (97% in 30 min) compared to adults (57% in 30 min) [39]. Since single ghrelin subcutaneous (sc) administration to mothers elevated fetal circulating ghrelin within 5 min [38], it is assumed that at least part of fetal ghrelin is derived from the maternal circulation. Additional studies investigating the sources of fetal ghrelin should follow.

#### 2.1.6. Placenta

Ghrelin mRNA and its acylating enzyme GOAT were detected to be expressed in the placenta [14,22]. In rats, during early pregnancy ghrelin is absent [40]. Its mRNA is detectable on embryonic day 12 and 17 [16], with a peak at day 16 and a decrease thereafter [40]. In detail, ghrelin is mainly expressed in the cytoplasm of labyrinth trophoblasts in rat placenta [40] as well as in the placenta of mice [41]. Noteworthy, under conditions of intra-uterine growth restriction in rats, placental ghrelin mRNA and peptide were elevated [42]. In the placenta of sheep, ghrelin was detected throughout the whole pregnancy with a peak at day 80 in maternal epithelium, caruncle, and trophectoderm [43]. In humans, ghrelin mRNA increased drastically due to decidualization of the endometrium [44], with dominant immunoreactivity in decidual cells and extravillous trophoblast cells [44]. Moreover, during the first trimester ghrelin was expressed predominately in placental cytotrophoblast cells and to a lesser extent in syncytiotrophoblast cells [40]. Further research is needed to understand the significance of ghrelin in placental functions.

#### 2.1.7. Umbilical Cord

Ghrelin is found in umbilical cord blood and is higher in the vein than in the arteries; thus, the placenta could be a source of fetal ghrelin [45]. Interestingly, levels of total as well as desacyl ghrelin in the umbilical cord correlate negatively with placental weight [45,46]. If this is related to placental insufficiency and intrauterine growth restriction (IUGR) warrants further research.

Although ghrelin was consistently detected in umbilical cord blood, its relation to maternal characteristics is unclear. While it was observed in one investigation that maternal plasma acyl ghrelin was higher than acyl ghrelin concentration in the cord blood [47], another study reported a contrary finding [48], and a third study found no difference between ghrelin concentration in the umbilical cord blood and in maternal serum [49]. Similarly inconsistent, one study found a positive [48], while other studies found no [50] or negative [51] correlation between umbilical cord ghrelin and maternal circulating levels. Lastly, total ghrelin levels were not correlated but acyl ghrelin were positively correlated between maternal and umbilical cord levels [47]. However, since a negative correlation between oxidative stress biomarkers in cord or maternal blood with umbilical cord ghrelin level was found [52], inconsistencies in data could be due to differences in level of oxidative stress. Data regarding the relation between gestational age and ghrelin in umbilical cord blood is also inconsistent. There is data indicating no correlation between both [50], as well as data suggesting a negative [53] or positive [48] correlation between ghrelin in cord blood and gestational age.

Interestingly, the majority of studies report a negative correlation between cord blood ghrelin and birth weight or length [45,46,50,53,54,55]. Consequently, increased umbilical cord blood ghrelin levels were measured in small for gestational age (SGA) neonates [45,46,53] compared to large for gestational age (LGA) infants [56], in newborns with IUGR [50], as well as in growth-restricted infants compared to neonates appropriate for gestational age (AGA) [57]. In contrast, only one report showed a positive correlation between cord blood ghrelin and neonatal weight and length [51]. Unsurprisingly, birth weight was shown to be a determinant of umbilical cord ghrelin concentrations in infants [56]. In addition, a negative relation between ghrelin concentration in cord blood and head circumference was observed in girls but not boys in one study [55], in both in another report [53], while in a third study a positive correlation between cord blood ghrelin and head circumference was shown to be independent from sex [51]. In only boys and not girls, acyl ghrelin in the umbilical cord additionally correlated negatively with abdominal and thigh circumference [55]. However, there were also reports of decreased ghrelin in cord blood in SGA newborns [58] and preterm babies [48], as well as data suggesting no difference in umbilical cord concentrations ghrelin between LGA and SGA newborns [59], between AGA and SGA piglets [60], and between AGA and LGA preterm infants [61].

Taken together, although not consistent, the majority of studies indicate a negative relation between umbilical ghrelin and body weight with increased levels in lighter newborns. This data strongly indicates a role of ghrelin in growth and energy metabolism in fetuses and newborns; however, if this knowledge can be used for clinical obstetrical or pediatric interventions should be evaluated in the future.

#### 2.1.8. Breast and Breast Milk

Ghrelin was observed to be expressed in alveolar and ductal mammary epithelial cells during pregnancy, with high intensity of mRNA and peptide expression from day 90 to 120 days compared to early pregnancy in goats [62]. In addition, the mammary gland was shown to secret ghrelin [63]; thus, unsurprisingly ghrelin can be found in breast milk [63]. In milk, desacyl ghrelin is higher than acyl ghrelin [64,65]. Total ghrelin levels were reported to decrease over time; thus, foremilk had higher concentrations than hindmilk [66], but also in milk from the second month total ghrelin concentrations were higher than in milk from the fifth month [66]. However, while during breast feeding acyl ghrelin also decreased, resulting in lower ghrelin levels in hindmilk than in foremilk; with time acyl ghrelin increased in milk, with elevated levels in milk from the fifth month compared to the second [66]. Interestingly, there is data showing that breast milk ghrelin concentration is higher than in maternal and cord blood [49,63], but there are also studies that observed lower ghrelin concentrations in breast milk compared to maternal plasma levels [67,68], while another found no difference between ghrelin levels in colostrum and maternal serum with a positive correlation between those two values [49]. Noteworthy, absolute values were very different in these studies, ranging from 97 [67] to 2125 pg/mL [63] in breast milk; thus, more studies should be performed to identify the concentration of ghrelin in breast milk and the underlying mechanisms of its alterations over time.

Noteworthy, in breast milk of mothers with obese infants lower ghrelin concentrations were detected than in breast milk of mothers with normal weight newborns [69]. However, the relation between milk and plasma ghrelin concentration was not different in pre-term compared to term infants [68]. These observations indicate that body weight, rather than gestational age, affects ghrelin levels, further corroborating ghrelin’s role in newborn growth and energy metabolism.

#### 2.1.9. Uterus

Ghrelin mRNA and peptide were detected in the uterine endometrium [36]; however, in a rather low concentration in the absence of pregnancy [44], and expressed during day 3–21 of the estrous cycle [70]. In detail, ghrelin was expressed in glandular epithelial and stromal cells of the endometrium, with highest immunoreactivity during the secretory phase [71]. In addition, in human myometrium ghrelin was detected which was decreased during labor [72]. The underlying mechanisms responsible for this change should be identified in the future to illuminate ghrelin’s role in the uterus in detail.

### 2.2. Presence in Plasma 

#### 2.2.1. Pregnancy and Delivery

In rodents, at mid-pregnancy, such as on day 10 and 15 of pregnancy, plasma ghrelin was decreased [12,41], while on day 20 [12] and day 23 [41] it was increased compared to non-pregnant rats [12]. Comparison of fasting plasma ghrelin concentrations throughout pregnancy showed a decrease with time, resulting in the lowest ghrelin level by day 18 of pregnancy in one study [73], while in nonfasted rats an increase of circulating ghrelin throughout pregnancy was observed [13]; in both investigations there was a normalization of ghrelin concentration at parturition [73]. Noteworthily, one study showed no change in circulating ghrelin due to pregnancy [74]. During the 115 days of pregnancy in sows, the highest plasma acylated ghrelin concentration was measured at day 30; in addition, after delivery this concentration decreased [75].

In humans, data about circulating ghrelin levels are not completely consistent. One study observed no effect of pregnancy on plasma ghrelin levels [76], while several investigations showed high levels of ghrelin in week 18 [77] and during the 2nd trimester in general [78], with a decrease in ghrelin from second to third trimester [78,79], resulting in low levels of circulating ghrelin in late pregnancy [77]. Similarly, while one study showed no effect of delivery on plasma ghrelin levels, two others observed increased serum levels during the postpartum [77,80]. These inconsistent data warrant further research identifying factors responsible for differences in ghrelin levels, such as fetal characteristics.

The hypothesis that inconsistencies in ghrelin levels during pregnancy could be due to fetal features is corroborated by observations that second and third trimester circulating acyl ghrelin levels correlated positively with neonatal waist circumference, and that maternal acyl ghrelin during third trimester correlated negatively with percent total neonatal body fat [81]. Moreover, maternal acyl ghrelin was found to be a predictor of LGA fetuses [82], and IUGR was related to decreased maternal ghrelin in serum [83].

Another explanation for inconsistencies in ghrelin levels between different studies could be the variability in the methods of ghrelin measurement. For accurate assessment of ghrelin in blood, sample stabilization is crucial, because otherwise ghrelin is rapidly desacylated, therefore omitting stabilization can cause inaccuracy and thus inconsistencies [84].

#### 2.2.2. Lactation

Compared with non-lactating rats, circulating ghrelin in lactating rats decreased, as measured on day 3 and 8 [12], while no difference between lactating and non-lactating dams regarding circulating ghrelin was observed on day 15 postpartum [85]. Similarly, in lactating women acyl ghrelin was decreased, compared to non-lactating women [67,75]. Interestingly, throughout lactation an increase of fasting plasma ghrelin was observed, with highest levels at the end of lactation [73]. Altogether, this data indicates a decrease of circulating ghrelin during lactation with normalization of ghrelin blood levels over time; the reasons for this reduction in circulating ghrelin, which could be increased secretion into breast milk and thus loss with breast feeding, need to be further investigated.

#### 2.2.3. Neonatal Period

Plasma ghrelin was observed to not be independently correlated to gestational age [86] and thus preterm infants born before 34 gestational weeks displayed no difference regarding circulating acyl ghrelin compared to those born after 34 gestational weeks [87]. Similarly, in term and preterm neonates, ghrelin levels were not different from each other [86].

Interestingly, data indicated that female neonates have higher plasma ghrelin levels than male infants [88]. It could be hypothesized that this is due to weight differences between female and male newborns, since it was observed that SGA infants also displayed increased plasma ghrelin concentrations when compared to AGA newborns [89]. Additionally, in preterm SGA or growth-restricted infants, higher circulating ghrelin levels were measured than in AGA newborns [87]. Moreover, ghrelin was increased also in newborns with a birth weight lower than 1500 g compared to those with a birth weight over 1500 g [87]; thus, data from studies reporting a relation between gestational age and circulating ghrelin [87,90] could be biased by differences in weight. Nevertheless, there is also data suggesting no difference in circulating ghrelin between AGA and SGA piglets [60]. With the exception of one study, showing a positive correlation between ghrelin with birth weight and head circumference in premature babies [91], the majority of studies found a negative relation between neonatal circulating ghrelin and birth weight or length [86,87,89], supporting ghrelin’s importance in body weight regulation. The usefulness of ghrelin as a marker for fetal growth abnormalities should be further investigated.

#### 2.2.4. Hormonal Treatment

Testosterone administration in peripubertal boys decreased ghrelin [92]. Similarly, in men, testosterone enanthate plus oral progestin reduced total ghrelin levels [93], while oral progestin alone for 3 weeks increased total ghrelin levels [93]. Moreover, while GnRH analog treatment in girls with central precocious puberty reduced plasma ghrelin [94], priming with estrogen in peripubertal girls did not affect ghrelin [92]. More studies are needed examining the effect of hormonal supplementation on the ghrelin system to understand regulation of ghrelin secretion, for example during sexual maturation. 

### 2.3. Effects of Ghrelin on the Reproductive System

#### 2.3.1. Hypothalamus

There is controversy as to whether ghrelin is actually expressed in the brain, based on failure to detect ghrelin immunoreactivity using commercially purchased antibodies and the need for highly sensitive PCR methods to detect ghrelin mRNA in the brain [95]. However, since ghrelin was shown to be able to cross the blood–brain barrier, a centrally-mediated effect of ghrelin on the HPG axis is probable [96]. Early on it was shown that ghrelin has a modulatory effect on hypothalamic GnRH secretion in vitro [97]. Direct ghrelin incubation of hypothalamic tissue of 50-day old male rats increased GnRH interpulse interval, while GnRH interpulse interval was reduced due to ghrelin incubation in hypothalami of 15-day old male rats via a NPY-Y5 receptor and partial MC3/MC4 receptor-dependent mechanism [98]. When male rats received intraperitoneal (ip) injections of ghrelin, their hypothalamic neurons displayed increased GnRH secretion interpulse intervals, independent of the rat’s age [98].

In peripubertal lambs, ghrelin injections increased the pulse amplitude for gonadotrophin release [99]. In addition, in female rodents it was shown that the estrous cycle affects ghrelin-induced firing modulation, since only GnRH-positive neurons in metestrus, not in proestrus, decreased firing rate and burst frequency in response to ghrelin [100]. It is supposed that this cycle dependency is due to changes in estrogen levels, because in vitro the ghrelin-induced increase in Ca^2+^ content of GnRH cells was abolished by applying estradiol [100], and additionally 90% of GHS-positive cells in the anteroventral periventricular nucleus and PVN expressed estrogen receptor-α [101]. In contrast to the observations in GnRH cells, in ovariectomized mice the ghrelin induced depolarization of Kisspeptin (Kiss1) neurons in the ARC rose from 22% to 75% after estradiol supplementation [102], supposedly due to estradiol-induced increase of GHSR mRNA expression. However, since intracerebroventricularly (icv) injected ghrelin decreased hypothalamic mRNA and protein levels of 3β-hydroxysteroid dehydrogenase, an enzyme of the steroid metabolism [103], the estradiol-induced enhancement of ghrelin effects could be opposed by a ghrelin-induced inhibitory effect on estradiol metabolism; however, this warrants further research.

#### 2.3.2. Pituitary

##### Follicle-Stimulating Hormone

The results observed after ghrelin administration on follicle-stimulating hormone (FSH) are not completely consistent. On one hand, in healthy young men acute infusion with acyl ghrelin had no effect on FSH secretion [104]. On the other hand, in proestrus afternoon and metestrus ghrelin stimulated FSH secretion from pituitary tissue in vitro [97], 3 day long ghrelin administration in prepubertal lambs increased FSHβ mRNA expression, FSH accumulation, and FSH serum levels [99], and similarly, 16 week long treatment with desacyl ghrelin elevated FSH in plasma [105]. This data points towards a dependency of ghrelin-induced FSH stimulation on timing and duration of administration. This hypothesis is further supported by reports of ghrelin-induced FSH inhibition e.g., at estrus GnRH-induced FSH secretion in vitro was reduced by ghrelin [97]. In vivo four consecutive intravenous (iv) ghrelin injections in ewes also reduced the GnRH-induced FSH surge [106]. In addition, in male adult rats twice daily sc injections throughout puberty of acyl or desacyl ghrelin also reduced plasma FSH [107]; in female mice intracerebroventricular injections and in women, peripheral acute injections also reduced the secretion of FSH [103,108]. Finally, in cows repeated iv ghrelin injection also decreased the area under the curve for FSH accompanied by reduced duration of the following estrous cycle [109]. Altogether, sex, species, timing, and duration of ghrelin application affect ghrelin’s action on FSH modulation.

##### Luteinizing Hormone

Few studies show no effect of acute acyl ghrelin injection in men on LH [104] or an increasing effect on LH in vitro on proestrus afternoon and metestrus [97] and in vivo after 16 week long treatment [105], while the majority of reports describe an inhibitory effect of ghrelin on LH. In expression studies, third ventricle ghrelin injection in rats decreased LH β-subunit gene expression [110]. In vitro, ghrelin inhibited GnRH-induced LH secretion throughout the estrous cycle [97], and also in vitro icv-injected ghrelin inhibited LH secretion throughout the estrous cycle [97].

Moreover, ghrelin was also able to suppress LH secretion in prepubertal and gonadectomized rats [111]. Interestingly, ghrelin infusion in ovariectomized monkeys reduced LH pulse frequency but not LH pulse in a corticotropin-releasing factor (CRF) dependent manner [112]. In contrast, ovariectomized rats treated with estradiol also displayed suppressed pulsatile LH secretions for about 1 h due to ghrelin but with reduced pulse frequency [113]. In healthy men iv ghrelin administration reduced LH peak levels, with prolonged intervals between pulses and less frequent pulses [104,114]. Similar effects were observed in women [108]; in addition, the area under the curve for LH secretion was also reduced [108]. Interestingly, ghrelin-induced reduction in LH pulse frequency was more pronounced under fasting conditions [115]. Ghrelin was also able to reduce GnRH-induced [106] or kisspeptin-10-induced LH secretion [107], and even under conditions of chronically elevated gonadotropin levels iv ghrelin infusion decreased LH concentrations [107].

In several other studies it was observed that circulating LH concentrations were reduced due to ghrelin administration in form of chronic sc injection of acyl or desacyl ghrelin in pubertal, adult [107] or food-restricted male rats [116], acute sc injection of acyl or desacyl ghrelin [107], bolus injection into the third cerebral ventricle of female sheep [117], 42 day long hypothalamic infusion in male mice [118], acute icv injection in female mice [103], as well as acyl ghrelin infusion in healthy men [104].

In male rats, chronic sc injection of acyl or desacyl ghrelin even delayed balanopreputial separation [107], and in heifers repeated iv ghrelin injection reduced the area under the curve for FSH leading to a reduced duration of the following estrous cycle [109]. These studies should be completed by investigations in ghrelin knock-out (KO) models or under conditions of acute ghrelin inhibition, to evaluate the endogenous role of ghrelin in sexual maturation and estrous cycle modulation.

#### 2.3.3. Testis

##### Testosterone Secretion

In hypogonadal men circulating ghrelin levels were decreased [119,120], and testosterone replacement for 6 months normalized circulating ghrelin [119]. Moreover, although in hypogonadal men plasma ghrelin correlated positively with total and free testosterone concentrations [119], in healthy men peripherally applied ghrelin was associated with reduced mean plasma testosterone [114], and serum ghrelin and testosterone correlated negatively with each other also in food restricted rats [121]. The relationship between circulating ghrelin and testosterone needs to be further investigated, since data so far is inconsistent.

In vitro ghrelin treatment of testicular tissue was in turn shown to have an inhibitory effect on testosterone secretion. In vitro ghrelin incubation of testicular slices additonally dose-dependently reduced basal and human choriogonadotropin (hCG)-induced testosterone secretion [122]; similarly, ghrelin inhibited human CG- and cAMP-stimulated testosterone secretion in vitro [17]. This inhibition was accompanied by reduced expression levels of mRNAs encoding steroid acute regulatory protein, P450 cholesterol side-chain cleavage, 3-beta-hydroxy steroid dehydrogenase, and 17-beta-hydroxy steroid dehydrogenase type III enzymes [17]; moreover, the inhibition of testosterone current was shown to be GHS-R1a-dependent and mediated via phospholipase C and protein kinase C [123].

In vivo, while acute icv injection of ghrelin decreased testosterone in serum of adult male rats [124], 16-week long treatment with desacyl ghrelin in rats increased testosterone in plasma [105], pointing to a duration-dependent effect of ghrelin on testosterone secretion.

##### Testicular Morphology

Systemic repeated ghrelin injection reduced testis weight in male rats [116], which could be due to increased apoptosis, since ghrelin was also shown to increase bax level in the spermatocytes and reduce proliferation-associated peptide in the spermatocytes as well as spermatogonia; thus, ghrelin may stimulate apoptosis [125]. Indeed, 10-day long daily sc ghrelin injection decreased different cell types (except for spermatogonia), seminiferous tubules diameter and their germinal epithelium thickness [126]. Additionally, 42-day long treatment with ip injected ghrelin even led to reduced sperm concentration, motility, percentage of spermatogonia and sperm [118]. On the cellular level, sc injected ghrelin increased vacuolated mitochondria, reduced endoplasmic reticulum and intracellular organelles in the testis, and caused several detachment areas between cell membrane and its basement membrane [126]. When injected directly into the testis, ghrelin reduced the proliferative activity of differentiating immature Leydig cells accompanied by reduced stem cell factor [127]. In Sertoli cells, physiological levels of ghrelin decreased glucose consumption and mitochondrial membrane potential, alanine, and acetate production in vitro [128]. The significance of the pro-apoptotic and anti-proliferative effects of ghrelin on testicular tissue should be identified in future investigations.

Since sc injections of ghrelin over 10 days increased glutathione peroxidase activity and decreased malondialdehyde levels, a biomarker for oxidative stress, in rat testis [129] it was suggested that ghrelin protects against cell stress; thus, several studies investigated ghrelin’s effect on testicular injury.

##### Testicular Injury

Testicular stress was shown to modulate the ghrelin system e.g., radiation increased ghrelin expression in the nucleus of differentiating spermatogonia [130] and testicular torsion increased testicular ghrelin expression [131], further supporting a role of ghrelin in testicular injury. Cryptorchidism-induced testicular weight change was improved by ghrelin treatment, accompanied by restoration of spermatogenesis and seminiferous tubule diameter [132]. In addition, ghrelin stimulated glutathione peroxidase activity, glutathione content, and catalase activity, while reducing thiobarbituric-acid-reactive substance concentrations as well as formation of giant cells and tubular vacuolization under conditions of cryptorchidism [133]. Similar effects on oxidative stress were observed in varicoceles, with increased levels of superoxide dismutase, sperm count and viability as well as decreased levels of malondialdehyde (MDA) and improved catalase activity due to ghrelin [134]. Ghrelin was similarly able to reduce HFD-induced testicular oxidative stress and activation of cleaved caspase-3, increasing testicular testosterone [105]. Additionally, in a testicular ischemia-reperfusion model, ghrelin administration reduced MDA values and increased activity of glutathione peroxidase as well as sperm motility, movement and concentration in the testis of rats [135]. Additionally, morphological ischemia-reperfusion induced changes were partially improved by ip injected ghrelin [136]. Ghrelin also had protective effects in chemotherapy-induced oxidative stress, e.g., it normalized cyclophosphamide-induced reduction of total antioxidant capacity and elevation of MDA, resulting in reduced abnormal sperm and increased number of spermatozoa and viability [137].

Similarly, cisplatin-induced decreases in testicular weight, sperm count, and motility were prevented by ip ghrelin administration [138]. Ghrelin also improved cisplatin-induced damage to the seminiferous epithelium [139] and increased cisplatin-induced reduced body and epididymal weights [139]. Overall, ghrelin acted anti-apoptotic by improving repair of DNA double stranded breaks via expression of gamma-H2AX, ataxia telangiectasia mutated and p53 [138], leading to reduced testicular cell death [139]. This is further corroborated by observations showing that ghrelin’s inhibition increased radiation-induced apoptosis of spermatogonia [130].

Ghrelin also showed positive effects on heat-induced testicular injury, by partially improving reductions of seminiferous tubules, Sertoli cell nucleus diameters, germinal epithelium height, miotic index, spermatogenesis rate, presence of spermatocytes, and volume densities after sc injection [140]. There is evidence for ghrelin-induced acceleration of testicular regeneration indicated by reduced expression of Bax, a pro-apoptotic protein in spermatocytes and increased number of proliferating-cell-nuclear-antigen immunolabeling cells due to sc ghrelin injection [141].

These observations stand in contrast to data indicating pro-apoptotic effects of ghrelin. Whether this is due to differences under basal and stress-induced conditions or other so far unidentified factors, e.g., timing and duration of ghrelin administration, should be investigated in further research. Since ghrelin was able to reduce expression of tumor necrose factor (TNF)-α, interleukin (IL)-1β, IL-6, IL-10, Toll-like receptor 4, and nuclear factor κB in testis under stress conditions [142], inflammatory factors could also be responsible for ghrelin-induced anti-apoptotic effects.

#### 2.3.4. Ovary

##### Ovarian Follicular Cells

In cultured granulosa and theca cells ghrelin incubation increased protein expression and cell proliferation [143] in a GHS-R type 1a dependent manner [144]. In detail, ghrelin increased bax accumulation, expression of bcl-2 [145], phospho-ERK 1/2 levels and PI-3 kinase activity [146] and decreased caspase-3 activity [147] and other markers of apoptosis [143]. In addition, ghrelin inhibited expression of the pro-apoptotic mediator MAP3K5 in porcine ovary [148]. The importance of ghrelin for optimal ovarian maturation is further supported by observations in GOAT knockout mice [149]. GOAT is responsible for acylating ghrelin, thus in the KO model no acyl ghrelin but only high levels of desacyl ghrelin were present [149]. The inability of ghrelin acylation was associated with a diminished number of small follicles especially primordial follicles resulting in an overall reduced number of ovarian follicles [149]. Moreover, observed ovarian transcriptome alterations related to the KO model could be responsible for premature ovarian development [149], indicating that the presence of acyl ghrelin may improve ovarian development. Furthermore, in cisplatin-induced ovarian failure, ip injected ghrelin almost normalized the number of primordial follicles [150].

In contrast, acute and 6-day long sc injection of rats reduced the mean diameter of each follicle, luteal cells, theca layer and whole ovarian volume, with associated intracellular alterations indicating apoptosis and cell death [151]. In line with this, ghrelin receptor antagonism reduced stress-induced depletion of primordial follicles [152]. Further research should aim to identify the underlying mechanisms responsible for ghrelin-induced proliferation as well as ghrelin-stimulated apoptosis.

Moreover, icv ghrelin inhibited the mRNA and protein levels of steroidogenic acute regulatory protein, cytochrome P450 17A1, and 3β-hydroxysteroid dehydrogenase in the ovary [103], and further steroid pathway enzymes [153], indicating an effect on hormonal secretion as described in detail below.

##### Granulosa Cells

In isolated ovarian granulosa cells, on one hand ghrelin increased expression of proliferating-cell-nuclear-antigen (PCNA) [154,155,156,157] as well as of cyclin [154,157]. However, it was also observed that high concentrations of ghrelin led to decreased protein kinase (PK) A accumulation in cultured chicken ovarian granulosa cells [158]. On the other hand, ghrelin incubation decreased expression of caspase-3, bax, bcl-2 [154,156], terminal deoxynucleotidyl transferase [155] and p53 in granulosa cells [157]. Altogether, it can be concluded that ghrelin stimulates granulosa cell proliferation [33] and inhibits apoptosis [159]; however, this should be corroborated by additional investigations.

##### Oocytes

Acute and 6-day long sc injection of ghrelin in rats reduced mean diameter of oocyte and zona plucida and induced intracellular alteration indicating apoptosis and cell death [151]; similarly, ip injected ghrelin reduced the average oocyte diameter in female fish [160]. However, some authors interpreted the decrease in diameter in the context of an increased number of ovarian follicles as a sign of decreased maturation [151], while others suspected stimulated maturation of oocytes [160], but there were also reports observing no alteration of oocyte nuclear maturation due to ghrelin incubation of oocytes [161]. This gap in knowledge needs to be addressed e.g., by usage of KO animals to understand the endogenous role of ghrelin in oocyte maturation.

Moreover, a ghrelin analogue injected sc reduced ovulation rate [162], and ghrelin decreased meiotic resumption of oocytes [37], and suppressed oocyte maturation as well as stage IV germinal vesicle breakdown in vitro in zebrafish [163]. Inhibition of oocyte maturation was associated with reduced blastocyst yield, Akt1 phosphorylation rate as well as increased ERK1/2 [164]. Incubation of cumulus oocyte complexes for 24 h with ghrelin at a dose of 800 pg/mL induced over-maturation [165], while a concentration of 20–60 pM increased cumulus cell death, apoptosis, and DNA damage [161]. Altogether these observations give rise to an inhibitory effect of ghrelin on oocyte maturation in a dose-dependent manner. Experiments where physiological concentrations of ghrelin are applied could help to illuminate the endogenous significance of ghrelin.

##### Corpus Luteum

Ghrelin injected sc reduced the number of corpora lutea with decreased mean diameter of corpora lutea in rats [151]. Moreover, desacyl ghrelin reduced vascular endothelial growth factor (VEGF) release from midluteal phase human corpora lutea [166] and also basal and hypoxia-stimulated VEGF release was reduced due to ghrelin [167], indicating an inhibition of angiogenesis due to ghrelin in luteal cells. Since cytochrome P45011A1 and 3-beta-hydroxysteroid dehydrogenase mRNA expression were also decreased due to ghrelin incubation of luteal cells [168], an inhibitory effect of ghrelin on ovarian hormonal secretion can also be hypothesized. Moreover, ghrelin also increased TNF in mid equine corpus luteum [169], pointing towards a cytostatic effect on the corpus luteum.

##### Estrogen Secretion

Ghrelin incubation of ovarian follicular cells increased estradiol secretion [143,147,170] and aromatase activity [143,147] in a GHS-R-1a-dependent manner [144]. However, at high concentrations of 100 pg/mL and 500 pg/mL (the physiological concentration in follicular fluid is 20 pg/mL), ghrelin reduced the secretion of estradiol in the ovaries of mature pigs [153]. This reduction was observed to be PKA as well as mitogen-activated protein kinase (MAPK) dependent [158]. Not only the concentration, but also the length of ghrelin appears to affect its estrogen stimulating property, since ghrelin_1–18_ decreased estradiol secretion, while it was increased by ghrelin_1-5_ and ghrelin_1–28_ [145]. These studies need to be completed by investigations under conditions of ghrelin inhibition e.g., by using ghrelin antibodies, to be able to evaluate ghrelin’s endogenous role in estradiol secretion.

In granulosa cells ghrelin incubation reduced estradiol secretion [33]. Additionally, in human granulosa lutein cells ghrelin treatment led to reduced estradiol in a ghrelin receptor-1a dependent manner [171]. Thus, in vivo ghrelin treatment also led to reduced secretion of estradiol from granulosa cells [172].

However, overall, in vivo ghrelin increased estradiol secretion from ovaries in 1 year-old and 3–5-year-old minks [173]. Moreover, food-restriction-induced reduction of estradiol secretion from ovaries was increased after three days of intramuscular (im) ghrelin treatment in vivo [174], and additionally in birds, im injected ghrelin partially prevented food-deprivation-induced reductions of estradiol [174]. However, it appears as if the effect of ghrelin on estradiol is opposite when centrally applied than when peripherally applied, since 3 nmol ghrelin icv injected decreased serum estradiol throughout the cycle in rats [175].

##### Testosterone Secretion

In vitro and in vivo ghrelin was observed to increase ovarian testosterone secretion [176,177], in a PKA- and MAPK-dependent manner [158]. Furthermore, food-restriction-induced reduction in testosterone from the ovaries was increased by ghrelin treatment for 3 days [174]. However, at high concentrations of 100 pg/mL and 500 pg/mL (the physiological concentration in follicular fluid is 20 pg/mL), ghrelin reduced the secretion of testosterone in ovaries of mature pigs [153]. Additionally, in cultured chicken ovarian fragments, only chicken but not human ghrelin stimulated testosterone release [170].

In cultured ovarian granulosa cells, human ghrelin inhibited, while chicken ghrelin stimulated testosterone output [170]. Additionally, ghrelin treatment of cultured granulosa cells decreased testosterone [155]. In vivo ghrelin treatment led to reduced secretion of testosterone from granulosa cells [172]. However, ghrelin did not affect overall plasma testosterone [177]. In summary, data about ovarian testosterone secretion in response to ghrelin is quite inconsistent and should be investigated more in the future; especially the significance of dose and cell-type need to be examined.

##### Progesterone Secretion

Ovaries cultured with ghrelin displayed higher progesterone secretion [178], however at high concentrations of 100 pg/mL and 500 pg/mL ghrelin reduced the secretion of progesterone [153]. Similarly, ghrelin treatment of isolated granulosa cells decreased (high concentration) or increased (low concentration) progesterone secretion [155]. In granulosa lutein cells obtained from human follicular fluid ghrelin reduced progesterone concentrations in a GHS-R1a-dependent manner [171]. Additionally, in corpora lutea desacyl ghrelin reduced midluteal progesterone [166], and ghrelin reduced both basal [34,167,168,169] and hCG-stimulated progesterone release from luteal cells [167]. Overall, icv injected ghrelin decreased serum progesterone during metestrus [175]. Investigations in KO models are needed to understand if ghrelin is indeed necessary for regulation of progesterone secretion.

##### Prostaglandin (PG) Secretion

Ghrelin stimulated PGF and PGE in porcine ovary [148], additionally it increased progesterone secretion in ovarian follicular fragments [154]. Noteworthily, while ghrelin_1–18_ and a synthetic activator of GHS-R1a increased progesterone in vitro, ghrelin_1–5_ and ghrelin_1–28_ suppressed its secretion [145], pointing towards a peptide length dependency of ghrelin’s stimulatory effect. In addition, cell dependency could also be suspected, since in contrast to observations in ovarian follicles, in isolated luteal cells ghrelin reduced PGE [167] and increased PGF [167,169], and in vivo ghrelin treatment led to reduced secretion of PGF from granulosa cells [172]. Moreover, sexual maturity could also affect ghrelin’s action, since in ovaries from 1 year old minks ghrelin incubation inhibited PGF secretion and in the ovaries of 3–5 year old minks ghrelin inhibited PGE release, but lost the ability to affect PGF [173]. The identification of underlying mechanisms responsible for the modulation of ghrelin’s effect on PG secretion by its amino acid length, cell type, and sexual maturation is necessary to better understand ghrelin’s endogenous significance in ovarian function.

#### 2.3.5. Embryo Development and Implantation

Ghrelin analogue sc treatment in mice before mating and during the beginning of pregnancy decreased the percentage of females impregnated by each male [179]. These observations could be a result of various inhibitory effects of ghrelin on embryo development. Ghrelin decreased blastocyst formation rates [165], rates of cleavage and total cell number of blastocysts [180] at high doses (100–250 nm/mL), accompanied by a reduced inner cell mass and number of trophectoderm cells in blastocysts [36]. Moreover 100 nm ghrelin inhibited two-cell embryo development in vitro [36]. Also in vivo, 2–4 nmol/mice sc injected around ovulation delayed embryo development [181]. In addition, it was shown that ghrelin incubation decreased embryo quality, indicated by decreased inner cell mass and total cell number [161] and that ghrelin was negatively associated with the number of viable embryos [182]. Moreover, follicles with lower ghrelin content produced embryos with successful cleavage and viable morphology compared to follicles displaying a higher ghrelin content [182].

However, at low doses (50 ng/mL) and very high doses (500 ng/mL) ghrelin incubation elevated blastocyst rates, total cell numbers per blastocyst [180,183] and cleavage rates [37]. Interestingly, it was observed that 800 pg/mL of ghrelin added to zygote cultures for 7 days reduced blastocyst production; however, when the medium was renewed daily the blastocyst formation increased, and ghrelin incubated embryos were shown to be of better quality than controls, suggested by downregulated expression of a gene related to embryo quality namely DNA methyltransferase 3 alpha [184], pointing towards an ameliorating effect of ghrelin on embryo development under certain circumstances and with careful consideration of doses. The endogenous effect of ghrelin on embryonic development and quality needs to be studied in KO models or conditions of acute ghrelin inhibition.

Regarding implantation, in vitro ghrelin induced cell proliferation and reduced caspase-3 activity and cell apoptosis in a human choriocarcinoma cell line [185]. Moreover, ghrelin decreased progesterone secretion from human choriocarcinoma cells [185] and increased prolactin, whose knockout leads to impaired fertility [186], in human endometrial stromal cells [71]. In addition, since ghrelin enhanced the decidualization of human endometrial stromal cells in vitro [44], a positive effect on implantation can be suspected, further corroborated by observations showing that in utero exposure to ghrelin deficiency resulted in 60% reduction in the rate of embryo implantation [187].

#### 2.3.6. Fetal and Neonatal Development 

Since desacyl and acyl ghrelin were shown to bind to fetal tissues by autoradiography [38], it was suggested that they play a role in fetal development. Indeed, in mice ghrelin analogue sc treatment before mating and during beginning of pregnancy decreased the ratio of number of fetuses per corpora lutea [179], and 2–4 nmol/mouse applied around copulation or implantation elevated the percentage of atrophied fetuses [181]. As a result, reduced follicular fluid ghrelin was related to higher pregnancy rates [188], indicating a negative effect of ghrelin on fetus quantity; however, other reports indicate a positive effect of ghrelin on fetus quality. This hypothesis is corroborated by observations of reduced number of pups born per litter, but no effect on number of successful pregnancies at term or gestational length due to daily sc ghrelin administration in the first half of pregnancy [189]. In addition, in rats acyl ghrelin and desacyl ghrelin stimulated proliferation of cultured fetal cells from skin, spinal cord and hypothalamus in the late stages of pregnancy [190]. Moreover, sc administration of a ghrelin analogue during pregnancy accelerated offspring maturation [162], and chronic ghrelin treatment stimulated fetal development resulting in increased birth weight, while immunization against ghrelin reduced birth weight [38]. In contrast, ghrelin administration suppressed weight gain of fetuses and dams during pregnancy when applied around implantation [181].

In vitro, incubation of neonatal cells from the spinal cord and hypothalamus with ghrelin resulted in stimulated proliferation [190]. In vivo, although immunoneutralization of ghrelin at a late stage of pregnancy did not affect survival or development including somatotropic function [16], in rabbits one-week long im ghrelin treatment before ovulation reduced pup mortality [155]. Moreover, increased plasma ghrelin concentration in pre-lambing ewes was related to improved lamb viability at 72 h after birth [90]. Since ip injected ghrelin into newborn rats from day 5 to 30 increased weight gain, while injection of rabbit anti-ghrelin antibody reduced weight gain [191], it might be hypothesized that the improved viability of newborns is due to ameliorated weight gain; however, this warrants further investigation. Whether ghrelin’s positive effects on fetal and neonatal development can be used in clinical approaches to improve fetal survival has to be further investigated.

#### 2.3.7. Delivery and Lactation

In biopsy specimens of human myometrium obtained at cesarean section ghrelin reduced spontaneous contractions and oxytocin-induced contractions in vitro [192], while in non-pregnant uterus of rats ghrelin increased power characteristics and shortened contraction and relaxation of smooth muscle in vitro [193].

In vitro ghrelin stimulated cell proliferation of goat mammary epithelial cells and elevated the expression of the proliferation-related peptides PCNA and cyclin B1, as well as prolactin [62]. Eight consecutive days of sc ghrelin treatment in nursing dams resulted in increased milk yield with elevated mammary casein mRNA expression and litter weight gain [194]. Interestingly, neither ip injected bromocriptine, sc applied haloperidol, nor icv administered oxytocin antagonist injection affected ghrelin in lactating dams [12]

#### 2.3.8. Sexual Maturation and Fertility

Immunoneutralization of ghrelin in rats at embryonic day 16 did not affect onset of puberty [16]; however, daily sc ghrelin injections in neonatal rats accelerated vaginal opening from day 30 to day 27 [195]. Similar effects were observed after endogenous ghrelin inhibition throughout pregnancy, namely earlier vaginal opening and increased ovarian volume indicating earlier sexual maturation [196]. In addition, fertility appears to be related to ghrelin, since mating of homozygote or heterozygote ghrelin KO female mice with homozygote ghrelin KO male mice led to a significantly reduced number of pups per litter [197]. In contrast, knockout of the ghrelin receptor showed no impact on reproductive performance [197]. Ghrelin absence showed various other long-lasting effects in the following generation: in adolescent male mice an earlier testis descent and increased relative testicular weight was observed, resulting in accelerated male puberty onset also accompanied by reduced relative testicular weight and sperm motility, without affecting fertility in adult males [196]. In turn, in adult female mice the prenatal treatment tended to increase the percentage of embryo loss and fetal atrophy [196]. Similarly, in utero ghrelin deficiency reduced fertility causing decreased litter size [187]. Conversely, in dairy cows increased plasma acyl ghrelin levels correlated with conception success and elevated total ghrelin levels were associated with successful conception from first insemination [198]. Moreover, 16 week long ip treatment with desacyl ghrelin in rats increased pregnancy rate and number of pups at birth in high fat diet-fed rats [105]. The clinical significance of exogenous ghrelin should be further evaluated.

Ghrelin is also able to affect sexual behavior, since third ventricle injection in rats elevated the number of mount, latencies to the first mount, intromission, ejaculation and post-ejaculatory interval, while decreasing the number of ejaculations [110]; however, ghrelin failed to induce penile erection [199].

### 2.4. Role of Ghrelin in Fertility- and Pregnancy-Related Health Conditions

#### 2.4.1. Polycystic Ovary Syndrome

Although ghrelin concentration in follicular fluid was not different in polycystic ovary syndrome (PCOS) compared to BMI-matched healthy controls [200], the majority of studies showed that in patients suffering from PCOS circulating ghrelin levels were decreased compared to healthy lean [201,202], obese healthy controls [201,203] and women with hyperandrogenemia [202]. Interestingly, obese adolescents with PCOS had lower ghrelin levels than lean ones [204]. Moreover, in patients suffering from insulin-resistant PCOS, circulating ghrelin was decreased in a similar extent as in patients that underwent a gastrectomy [201]. Additionally, since serum ghrelin was comparable to healthy controls in insulin-sensitive subjects with PCOS but not in insulin-resistant subjects with PCOS [201], it can be assumed that the extent of decrease of ghrelin in women with PCOS depends on body weight and the severity of PCOS; however, this warrants further research. 

Consequently, therapies improving PCOS were shown to increase circulating ghrelin levels e.g., metformin treatment of insulin-resistant subjects with PCOS increased ghrelin [201]. Moreover, antiandrogen flutamide oral treatment of women with PCOS for 6 months increased plasma ghrelin, with a negative correlation between the change of plasma ghrelin and change of plasma androgen and a positive correlation between plasma ghrelin changes and insulin sensitivity [205]. Interestingly, multiple regression showed that plasma ghrelin changes were mainly due to changes of androgen levels rather than improved insulin sensitivity in antiandrogen flutamide-treated subjects with PCOS [205]. This is further corroborated by investigations showing that ghrelin correlated negatively with PCOS-associated hyperandrogenemia [202,203].

In addition, ghrelin also correlated positively with anovulation and polycystic ovary morphology in subjects with PCOS [202] and negatively with BMI and insulin [206]. Noteworthy, ghrelin could also be involved in an increased susceptibility of developing PCOS, since frequency of the single nucleotide polymorphism SNP501A/C A allele in the promoter of the ghrelin gene was increased in women with PCOS and was associated with higher BMI than the CC allele [207]. If altered ghrelin levels are the cause or consequence of PCOS needs to be further investigated in the future.

#### 2.4.2. Hyperemesis Gravidarum

Data from women with hyperemesis gravidarum (HG) is not consistent. There are studies showing increased ghrelin in subjects with HG [208] for both acyl and total ghrelin [209]. Moreover, there is a report of reduced serum ghrelin levels in women with HG [210] or a decreased acyl to total ghrelin ratio among the patients with HG [209]. Finally, there are also investigations that found no difference regarding acyl or desacyl ghrelin between women with HG and healthy pregnant women [211,212].

#### 2.4.3. Pregnancy-Induced Hypertension and Preeclampsia

Several reports observed that plasma ghrelin concentrations correlated negatively with systemic blood pressure in normal and hypertensive pregnant women [213,214] and patients with preeclampsia [215], with a negative correlation between ghrelin and uterine artery doppler index values in subjects with preeclampsia [216]. Consequently, systolic blood pressure was shown to be independently associated with serum ghrelin [216] and ghrelin was reduced in patients with preeclampsia compared to normotensive pregnant patients [215,217].

Interestingly, one study demonstrated that while in early onset preeclampsia (<34 weeks) ghrelin was reduced, it was increased in late onset preeclampsia [218]. Variations of ghrelin levels throughout pregnancy could be a reason while in contrast to the data displayed above there are also reports of increased circulating ghrelin levels in pregnancy-induced hypertension [213] and in mild and severe preeclampsia compared to healthy controls [216]. In following studies, timing of ghrelin analysis needs to be paid attention to in order to clarify the significance of ghrelin changes throughout pregnancy in healthy individuals and those suffering from HG.

Nevertheless, since ghrelin activated the Jagged1/Notch2 pathway inducing increased VEGF, which is decreased in preeclampsia [218] a positive effect of ghrelin on hypertension in pregnancy is assumed. This appears to be restricted to conditions in utero, since cord blood of newborns exposed to gestational hypertension was not different regarding ghrelin levels compared to cord blood of newborns that were not exposed [53]. To be able to use this knowledge in the clinical setting in the future, ghrelin’s VEGF-stimulatory effect should be investigated in more detail.

#### 2.4.4. Gestational Diabetes Mellitus (GDM)

Various studies observed decreased circulating ghrelin in patients with GDM compared to non-diabetic pregnant women [65,219], in insulin-requiring subjects with GDM compared to non-diabetic and diet-requiring women with GDM [88], and in patients with GDM 2 days after parturition [64]. In line with these findings, increased fasting ghrelin was found to be associated with decreased odds of developing GDM in women with risk factors for GDM adjusted for maternal obesity [220]. In some studies, decreased ghrelin was found in breast milk [65] or colostrum [64]. Moreover, even ghrelin in the umbilical vein of newborns of mothers with diabetes was reduced compared to those without diabetes in pregnancy [54].

In contrast, in women with GDM placental ghrelin mRNA was more abundant than in healthy patients [221], serum ghrelin levels were elevated in mothers of LGA or AGA with GDM compared to healthy controls with AGA babies [82], and desacyl but not acyl ghrelin throughout a meal was elevated in women with GDM [221,222]. Noteworthy, 15 days after delivery ghrelin in serum and mature milk were similar between women with GDM and those without [64], pointing strongly towards a time-dependency of ghrelin in GDM.

Altogether, data indicates a decrease in circulating ghrelin due to GDM and a normalization after delivery. If ghrelin could be a marker of GDM and if it is implicated in an increased risk of diabetes mellitus after GDM should be studied in the future.

## 3. Nesfatin-1

### 3.1. Expression in Tissues Implicated in Reproduction

#### 3.1.1. Hypothalamus

NUCB2/nesfatin-1 was found to be intensely expressed in the mouse hypothalamus [223] as well as in murine hypothalamic cells [224]. In the nucleus lateralis tuberis posterioris and the nucleus anterior tuberis of goldfish and murine hypothalamic cells NUCB2/nesfatin-1 expression was colocalized with GnRH [224,225], giving rise to an implication in reproduction. This hypothesis was further corroborated by different expression patterns throughout sexual maturation. In detail, NUCB2 mRNA level in the hypothalamus was highest in infant (10 d) rats and lowest in prepubertal rats [226]; thus, there was a trend of decreasing hypothalamic NUCB2 mRNA expression throughout neonatal to pre-pubertal development (day 10–30) of male and female rats [227]. In addition, in adult male rats a moderate level of NUCB2 mRNA expression was noted [226].

Noteworthy, testosterone especially seems to have modulating effects on hypothalamic NUCB2/nesfatin-1 expression. While in a murine hypothalamic cell line testosterone increased NUCB2 protein and mRNA [224], incubation of hypothalamic tissue with testosterone resulted in decreased NUCB2 mRNA expression [223]. Similarly, testosterone treatment after castration of mice, which had no effect on hypothalamic NUCB2 mRNA expression, decreased expression of NUCB2 mRNA in the hypothalamus [223]. If there are other factors, especially hormones such as estrogen or progesterone that can modulate hypothalamic NUCB2 expression warrants further research.

#### 3.1.2. Pituitary

Autoradiography demonstrated binding of nesfatin-1 to the pituitary of rats [228]. In addition, expression of NUCB2 mRNA was also found in the pituitary [223,226,229] in a higher concentration than observed in other organs [230] as well as in murine pituitary (LβT2) cells [224]. The observation that NUCB2/nesfatin-1 protein was found in the anterior pituitary gland [230] further supports the hypothesis that nesfatin-1 is implicated in reproductive functions. Similar to expression in the hypothalamus, the expression of NUCB2 mRNA was different depending on sexual maturation: it was highest in pubertal rats and adult male rats and lowest in prepubertal male rats [226].

In contrast to expression in the hypothalamus, mRNA NUCB2 expression in the pituitary was increased due to incubation with testosterone and, while castration of mice decreased NUCB2 mRNA expression it was increased after testosterone supplementation [223]. Additionally, in LβT2 cells testosterone increased NUCB2 protein [224]. In contrast, in vivo 2.5 days after ingestion of testosterone goldfish displayed decreased NUCB2 expression in the pituitary [9], indicating that testosterone increases expression of nesfatin-1 in the pituitary acutely, resulting in a compensatory decrease afterwards; however, this hypothesis should be tested by more investigations in the future.

In vitro, 17β-estradiol increased NUCB2 protein and mRNA in LβT2 cells [224], but also in cultured pituitary NUCB2 mRNA expression was elevated by estradiol [230]. In line with these observations, after ovariectomy NUCB2 mRNA expression in the pituitary was reduced and elevated after progesterone and estradiol injection [230]. In contrast, in vitro progesterone or progesterone with estradiol decreased NUCB2 mRNA expression in cultured pituitary [230]. In vivo, mRNA NUCB2 expression in the pituitary was decreased 2.5 days after ingestion of estradiol in goldfish [9]. These inconsistent data warrant further research.

#### 3.1.3. Testis

^125^I-nesfatin-1 autoradiography showed binding of nesfatin-1 to the testis of rats [228]. In mice, rat, poultry and human NUCB2 mRNA was expressed in testes [231,232,233]. In more detail, NUCB2/nesfatin-1 protein was expressed in interstitial mature Leydig cells in rats, human and mouse [226,232] and when Leydig cells were eliminated NUCB2/nesfatin-1 protein was found in Sertoli cells and Leydig cell progenitors in rats [232]. In addition, NUCB2/nesfatin-1 was expressed in the columnar epithelium of the epididymis in mice [229]. Noteworthy, the expression of NUCB2/nesfatin-1 was higher in testis and epididymis than in hypothalamus in mice [229]. NUCB2/nesfatin-1 protein in testis was increased by pituitary LH [226,232] and by aging [226,232]. In detail, testicular NUCB2/nesfatin-1 protein [226,232] as well as NUCB2 mRNA [226,232] increased from the puberty-to-adult transition. Other factors modulating testicular NUCB2/nesfatin-1 expression should be identified in future research.

#### 3.1.4. Ovary

NUCB2 mRNA and NUCB2/nesfatin-1 peptide were expressed in the ovaries of mice [234] and poultry [233]. Interestingly, murine nesfatin-1 expression was higher in ovary than in hypothalamus [229]. Ovarian expression of NUCB2/nesfatin-1 was highest during the estrus period [234] and was stimulated after pregnant mare serum gonadotropin (PMSG) ip administration [234]. Moreover, low NUCB2/nesfatin-1 immunoreactivity was detected in hyperactive ovary and shell gland [233], while NUCB2/nesfatin-1 immunoreactivity was high in regressed ovary (stromal cells) and shell gland (endometrium) [233], resulting in a negative correlation between ovarian activity and ovarian NUCB2/nesfatin-1 expression [233]. The functional importance of this correlation should be studied further.

On the cellular level, NUCB2/nesfatin-1 like immunoreactivity was detected in follicle cells of zebrafish and goldfish [225], in porcine theca and granulosa cells [235] as well as in murine interstitial cells of the ovary [229], but not in oocytes [225,235]. Regarding granulosa cells, NUCB2/nesfatin-1 was intensely expressed throughout all developmental stages [236]. Noteworthily, the number of transcripts decreased during transition from small follicles to larges follicles [235], and rats with letrozole induced-PCOS displayed reduced NUCB2 mRNA and protein expression in granulosa layer [237]. The mechanisms responsible for changes in nesfatin-1 due to follicular development and dysfunction need to be investigated in order to identify novel therapeutic approaches.

#### 3.1.5. Uterus and Placenta

NUCB2 mRNA and NUCB2/nesfatin-1 protein were expressed in the uterus of mice [234], where it was expressed in higher density than in the hypothalamus [229]. NUCB2/nesfatin-1 expression in the uterus was the highest during estrus [234] and increased due to proestrus [238]. Intraperitoneal PMSG administration, but not hCG injection, also increased NUCB2/nesfatin-1 immunoreactivity [238]. Moreover, the expression of NUCB2/nesfatin-1 was decreased due to ovariectomy and normalized after sc 17β-estradiol administration [238]. However, in humans there was no association between nesfatin-1 and uterine length or endometrial thickness [239]; thus, the significance of nesfatin-1 in the uterus is not fully understood yet and needs to be investigated in the future. On the cellular level, NUCB2/nesfatin-1 immunoreactivity was observed in epithelial cells of the endometrium [229], oviduct [238], and uterine glands [229], indicating an implication of nesfatin-1 in uterine secretion, which also warrants further research.

In addition, NUCB2/nesfatin-1 immunoreactivity was observed in amnion and decidua of the rat placenta [240]. In more detail, in mice NUCB2/nesfatin-1 was expressed in the ectoplacental cone as well as parietal trophoblast giant cells and early spongiotrophoblast from embryonic day (E) 7.5 to E9.5 [241]. From E10.5 to E12.5 NUCB2/nesfatin-1 expression was observed in the developing labyrinth, and from E12.5 to E17.5 NUCB2/nesfatin-1 was expressed in the glycogen trophoblast cells, syncytiotrophoblast, sinusoidal trophoblast giant cells, and fetal capillary endothelial cells of the labyrinth of mouse placenta [241]. In contrast, in human chorionic villi, NUCB2/nesfatin-1 was observed in syncytiotrophoblast throughout all trimesters and even increased with syncytialization of human primary trophoblast cells [241]. Since syncytiotrophoblasts are implicated in establishing nutrient circulation between the embryo and mother, this observation could indicate a role of nesfatin-1 in intrauterine nutrition and growth; however, this assumption needs to be corroborated by future studies. In addition, the reasons for inter-species differences should be addressed.

#### 3.1.6. Cord Blood and Breast Milk

Nesfatin-1 peptide found in cord blood correlated positively with maternal serum concentration and negatively with gestational age [242]. Nesfatin-1 peptide was also detected in breast milk, with higher concentrations in mature milk than in colostrum [65]. More investigations analyzing umbilical cord blood and breast milk are needed to better understand nesfatin-1′s role in reproduction especially in fetal and newborn nutrition and growth.

### 3.2. Presence in Plasma 

Nesfatin-1 peptide in blood was shown to positively correlate with FSH, estradiol and progesterone, and negatively with LH, and total testosterone in female rats [237]. However, in girls with central precocious puberty, nesfatin-1 peptide serum levels did not correlate with gonadotropin or estradiol levels [239], indicating a need for further research to identify the hormones able to modulate nesfatin-1 levels, as well as the underlying mechanisms.

Moreover, although in rats the lowest serum nesfatin-1 peptide concentration was detected in prepubertal males, there was no difference between prepubertal and adult rats regarding circulating nesfatin-1 peptide levels [226]. Similarly, in girls with central precocious puberty nesfatin-1 peptide serum levels were not different from controls [239] and there was no association between nesfatin-1 peptide plasma level and menopausal status [243], suggesting no significant effect of sexual development on circulating nesfatin-1.

Nevertheless, an implication of nesfatin-1 in reproductive function cannot be excluded, since throughout pregnancy in rats significant variations in serum nesfatin-1 protein were detected; in detail, from gestational days 12 to 16 and 21 circulating nesfatin-1 peptide decreased [240]. In addition, nesfatin-1 hormone plasma level increased in the months after spawning [244]. Moreover, rats with letrozole induced-PCOS displayed reduced nesfatin-1 peptide in their blood [237], while in humans with PCOS circulating nesfatin-1 peptide was increased compared to healthy age-matched controls [245]. More studies investigating nesfatin-1 throughout sexual maturation, in pregnancy, and during the postpartum period should follow to corroborate or refute current data.

### 3.3. Effects of Nesfatin-1 on the Reproductive System

#### 3.3.1. Hypothalamus

In vitro, incubation of murine hypothalamic cells with nesfatin-1 elevated mRNA expression of Kiss1 receptor and GnRH as well as of GnRH protein after 6 h [224]. In contrast, in vivo nesfatin-1 led to reduced hypothalamic expression levels e.g., icv injected nesfatin-1 reduced expression of hypothalamic GnRH and kisspeptin mRNA [226]. Similarly, acute ip injected nesfatin-1 reduced (75%) hypothalamic GnRH-II and GnRH mRNA 15 min post-injection in goldfish [225]. Moreover, ip injection of synthetic as well as nesfatin-1-like peptide also reduced hypothalamic mRNA expression of GnRH and brain aromatase in male and female goldfish [246]. In addition, nesfatin-1-like peptide reduced mRNA expression in the hypothalamus of gonadotropin-inhibiting hormone, of its receptor and kisspeptin in male and female goldfish, while synthetic nesfatin-1 additionally decreased the hypothalamic mRNA expression of the kisspeptin receptor within 15 min in male and female goldfish [246]. Identifying the underlying mechanism responsible for differences in vitro and in vivo examinations could help to better understand the role of nesfatin-1 in hypothalamic functioning.

#### 3.3.2. Pituitary

In vitro incubation of murine pituitary cells with nesfatin-1 elevated LHβ mRNA and protein expression after 6 h [224]. In contrast, in vivo acute icv injected nesfatin-1 reduced LHβ and FSHβ mRNA in the pituitary [226]. The same effect on gene expression in the pituitary was observed 60 min after ip injection of nesfatin-1 in goldfish [225]. Similarly, ip injected nesfatin-1-like peptide reduced mRNA expression in the pituitary of LHβ, FSHβ, kisspeptin and its receptor, while increasing mRNA expression of gonadotropin-inhibiting hormone and of its receptor in male and female goldfish [246]. Additionally, ip injection of synthetic nesfatin-1 reduced mRNA expression of pituitary LHβ and FSHβ within 60 min and kisspeptin receptor within 15 min in male and female goldfish [247]. The effects of nesfatin-1-induced suppression of LH expression, e.g., on estrous cycle, sexual maturation, fertility etc., should be investigated in the future.

#### 3.3.3. Testis

In vitro nesfatin-1 incubation of testis increased basal [226,231] and hCG-stimulated testosterone secretion [232]. Additionally, incubation of Leydig cells with nesfatin-1 increased expression of 3β-hydroxysteroid dehydrogenase (3β-HSD), 17β-hydroxysteroid dehydrogenase (17β-HSD) steroidogenic acute regulatory (*StAR*) and cytochrome P450 cleavage (P450scc) mRNA [226]. Moreover, nesfatin-1 testicular incubation stimulated spermatogenesis by increasing PCNA and bcl2 and decreasing caspase-1 and nitric oxide, promoting cell proliferation and survival as well as suppressing apoptosis and oxidative stress in testis [231]. Nesfatin-1 incubation had a positive effect on energy metabolism in testis by increasing the expression of steroidogenic markers, of insulin receptor proteins, and of GLUT8 proteins, leading to stimulated intra-testicular transport of glucose and production of lactate in the testes [231].

In vivo, acute icv injected nesfatin-1 increased the expression of genes of 3β-HSD, 17β-HSD and P450scc in pubertal rats’ testis, while it was decreased in adult rats [226]. In both pubertal and adult rats, icv nesfatin-1 reduced testicular *StAR* gene expression [226], indicating age-dependent effects of nesfatin-1 on steroid metabolism. Furthermore, nesfatin-1-like peptide and synthetic nesfatin-1 affected gonadal expression activity, e.g., ip injection of nesfatin-1-like peptide into goldfish reduced mRNA expression in the testis of LH and FSH receptor, gonadotropin-inhibiting hormone and its receptor as well as kisspeptin [246]. Moreover, synthetic nesfatin-1 reduced LH and FHS receptor mRNA and increased gonadotropin-inhibiting hormone, gonadotropin-inhibiting hormone receptor, kisspeptin 1 and kisspeptin receptor gonadal mRNA in male goldfish [247].

Chronic ip treatment with nesfatin-1 in vivo had various effects on gonadal function in pubertal male mice [248]. Firstly, it increased the expression of proteins involved in steroid hormone production including LH-R, *StAR*, P450scc, 3β-HSD and 17β-HSD proteins as well as markers of spermatogenesis such as bcl2, inducing cell survival and PCNA promoting proliferation [248]. These changes resulted in stimulation of testis maturation [248]. Moreover, this treatment stimulated the expression of testicular super oxide dismutase, catalase, and glutathione peroxidase enzyme activities, leading to decreased oxidative stress [248]. In addition, chronic nesfatin-1 treatment increased the expression of insulin receptor and GLUT8 proteins and in intra-testicular glucose concentration and LDH activity in pubertal mice [248,249], which could be responsible for enhanced transport of energy substrates ameliorating spermatogenesis and steroidogenesis; however, this warrants further research. These positive effect of nesfatin-1 on proliferation, the antioxidant system and energy metabolism can also be observed under conditions of testicular dysfunction, e.g., in T2DM [249], after testicular torsion [250], and nicotine exposure [251].

In the future, the endogenous role of nesfatin-1 in testicular functions could be further investigated using KO models or acute nesfatin-1 antagonists.

#### 3.3.4. Ovary

In vitro, nesfatin-1 exerted an inhibitory effect on zebrafish reproduction by reducing basal germinal vesicle breakdown during the oocyte maturation and inhibiting stimulation of maturation-inducing hormone on germinal vesicle breakdown. [225]. Similar observations were made in zebrafish oocytes after incubation with nesfatin-1-like peptide resulting in reduced oocyte maturation [246]. In contrast, in porcine oocytes nesfatin-1 stimulated both the cleavage and blastocyst rate of activated oocytes from small follicles, without affecting meiotic maturation and development of oocytes from large follicles [235]. However, in porcine oocytes incubated in a medium without pyruvate, meiotic maturation of oocytes was improved by nesfatin-1 [235].

In vivo, ip injection of nesfatin-1-like peptide reduced mRNA expression in the ovary of FSH receptor, gonadotropin-inhibiting hormone and its receptor and kisspeptin in goldfish [246]. Similarly, synthetic nesfatin-1 reduced ovarian mRNA expression of the LH and FSH receptors in goldfish after ip injection; however, in contrast to nesfatin-1-like peptide, synthetic nesfatin-1 increased ovarian mRNA expression of gonadotropin inhibiting hormone, its receptor as well as kisspeptin 1 and its receptor in goldfish [247]. In granulosa cells, nesfatin-1 in vitro increased progesterone secretion as well as cell proliferation via modification of nitric oxide production and non-enzyme scavenging activity [236].

#### 3.3.5. Plasma and Serum

Icv injected nesfatin-1 in a dose of 200 pmol had no effect on circulating GnRH in male rats; however, it increased plasma FSH and LH [252]. In contrast, in prepubertal and adult rats icv injection of 10 μg nesfatin-1 decreased FSH and LH concentrations in serum [226]. Similarly, acute ip injection of 50 ngnesfatin-1 per gramm of body weight reduced LHβ 60 min post injection in goldfish [225].

In line with the observations above, 200 pmol nesfatin-1 icv injected in male rats elevated plasma testosterone [252], while icv injection with 10 μg nesfatin-1 decreased serum testosterone in prepubertal and adult rats [226]. Similarly, also ip injection of nesfatin-1-like peptide and synthetic nesfatin-1 reduced plasma testosterone in female and male goldfish [246,247]. In addition, plasma estradiol in female and male goldfish was also decreased due to ip injection of nesfatin-1-like peptide or synthetic nesfatin-1 [246,247].

### 3.4. Role of Nesfatin-1 in Fertility- and Pregnancy-Related Health Conditions

#### 3.4.1. Polycystic Ovary Syndrome

In the present literature, two studies report an increased circulating level of nesfatin-1 peptide in patients suffering from PCOS compared to controls [245,253], with strong positive correlations between nesfatin-1 and BMI and HOMA-IR [245,253]. In contrast, in two other studies decreased circulating nesfatin-1 peptide was observed in patients with PCOS with no or a negative correlation to BMI and HOMA-IR [254,255]. Altogether, inconsistent results clearly suggest the need for further investigation.

#### 3.4.2. Hyperemesis Gravidarum

The observations of circulating nesfatin-1 peptide in patients with HG were similarly inconsistent: while one study reported no difference regarding nesfatin-1 peptide between patients with HG with lower BMI and controls [210], another investigation found an increased nesfatin-1 protein blood concentration in patients with HG matched for age, BMI and pregnancy development compared to controls [211]. These different results underline the importance of matching and the necessity of more studies in patients with HG.

#### 3.4.3. Pregnancy-Induced Hypertension and Preeclampsia

Although circulating nesfatin-1 peptide was shown to be positively correlated to systolic and diastolic blood pressure in women [253], there is no direct relationship between circulating nesfatin-1 protein and preeclampsia. In one study patients with preeclampsia displayed decreased circulating nesfatin-1 protein levels [256], while in another report nesfatin-1 peptide levels were increased in patients with preeclampsia compared to normotensive individuals [217]. Since there was no difference regarding circulating nesfatin-1 protein between obese and non-obese patients with preeclampsia [217], other factors influencing nesfatin-1 levels in subjects with preeclampsia need to be identified in future studies.

#### 3.4.4. Gestational Diabetes Mellitus (GDM)

A majority of studies found reduced circulating nesfatin-1 protein in patients with GDM [65,242,257,258,259], and even decreased nesfatin-1 peptide concentrations in milk of subjects with GDM [65]. In contrast, there are also reports about increased levels of NUCB2/nesfatin-1 peptide in serum in patients suffering from GDM [260] and in the cord blood of babies born to mothers with GDM [260,261], resulting in nesfatin-1 being an independent risk factor for GDM [260].

The influencing factors responsible for differences in nesfatin-1 between patients with and without GDM are not completely clear. While some report no correlation between nesfatin-1 peptide level in serum and BMI, insulin sensitivity, fasting glucose and maternal age [242,258], gestational age [258] as well as circulating insulin [261], others observed a negative correlation between nesfatin-1 peptide and weight, BMI, fasting glucose and insulin sensitivity [257]. Moreover, there are investigations showing a positive correlation between circulating nesfatin-1 protein and gestational age [258], insulin and BMI [260]. These inconsistencies underline that more studies are needed examining nesfatin-1 levels in women with GDM and associated factors.

Examinations of cord blood showed no difference in nesfatin-1 peptide between newborns of mothers with or without diabetes [242], and in addition there was no correlation between nesfatin-1 protein and glucose [242] or insulin levels in umbilical cord blood [242,261]. Thus, it appears that altered maternal nesfatin-1 levels in subjects with GDM do not affect fetal circulating nesfatin-1; however, this hypothesis warrants further investigation.

#### 3.4.5. Abnormal Birth Weight

While nesfatin-1 peptide umbilical cord blood levels were decreased in LGA newborns compared to AGA infants [261], circulating NUCB2/nesfatin-1 in SGA and IUGR babies was increased compared to normal weight neonates [262,263], even 7 and 28 days after birth [262]. As a consequence, there was a negative correlation between NUCB2/nesfatin-1 and oral caloric intake in SGA newborns [262] and fetal birth weight in IUGR newborns [263], as well as a positive correlation with insulin resistance in SGA [262]. The mechanisms involved in nesfatin-1-related fetal growth modulation need to be identified to assess their clinical importance in order to find new approaches to address fetal and neonatal growth restriction.

## 4. Summary

### 4.1. Ghrelin 

#### 4.1.1. Ghrelin’s Expression and Effects along the HPG Axis

**Figure 1:**
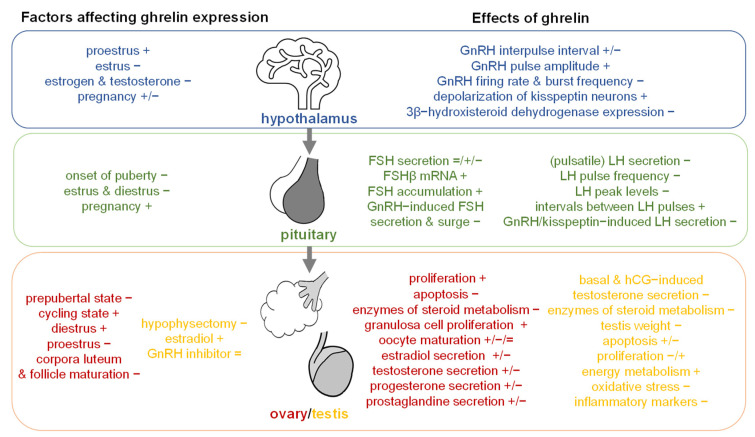
Ghrelin’s role along the HPG-axis. + increasing/stimulating or increased/stimulated; − reducing/inhibiting or reduced/ inhibited; = not affecting/not changing or not affected/unchanged; HPG hypothalamic-pituitary-gonadal; R receptor; FSH follicle stimulating factor; LH luteinizing hormone; GnRH gonadotropin-releasing hormone.

Ghrelin expression in the hypothalamus [1] is modulated by hormonal changes [9]; thus, varies throughout the estrus cycle [10,11] and pregnancy [12,13]. In turn, ghrelin has an effect on hypothalamic GnRH pulses in vivo and vitro [98] in an estrogen and estrogen receptor-α- [101,102] and estrous cycle-dependent fashion [100] (Figure 1).

Ghrelin’s expression in the pituitary is higher compared to most other tissues [14,15] and is affected by sexual maturation [16] and pregnancy [13]. Conversely, ghrelin was shown to have a stimulatory effect on FSH expression [97,99,105] as well as an inhibitory effect on GnRH-induced FSH secretion [97,106,107], depending on timing and duration of ghrelin administration. On LH expression and GnRH-induced LH secretion, ghrelin has a more consistent inhibitory effect [97,104,106,110,114], also reducing circulating LH levels [104,107]. As a result, chronic ghrelin treatment is able to modulate pubertal maturation [107,195] (Figure 1).

Ghrelin is also expressed in the testis [14], predominately in Leydig cells [18] as well as in sperm and their precursor [20,21]. Ghrelin’s testicular mRNA and protein levels are modulated by LH [19], estradiol [23], and testosterone [24], whereas ghrelin has an inhibitory effect on testicular testosterone secretion [17,122] via suppression of expression of enzymes crucial for steroid metabolism [17] after acute but not chronic application [105]. In addition, ghrelin increases apoptosis and reduces proliferation in spermatocytes [125] and Leydig cells [127]. Ghrelin’s expression in the testis is affected by testicular stress [131]; in turn, ghrelin normalizes stress-induced testicular morphological [132,136], oxidative stress [133,135], and inflammatory changes [142] (Figure 1).

Ghrelin mRNA and peptide are both expressed in the ovary [14,15] and are greatly affected by the estrous cycle [27]. The highest ovarian expression of ghrelin was consequently observed during diestrus and the lowest during proestrus [10,27]. Noteworthy, ghrelin has various effects on ovarian morphology and function: firstly, in ovarian follicular cells ghrelin inhibits apoptosis [147,148] and stimulates cell proliferation [143]. Secondly, ghrelin decreases the diameter of oocytes and increases number of ovarian follicles [151], probably resulting in suppressed oocyte maturation [163]. Thirdly, ghrelin suppresses steroid pathway enzymes [153] resulting in altered hormonal secretion, such as of progesterone [155,175], estrogen [33,143,147,153,170], and testosterone [153,155,176,177] in a dose- and cell-type dependent manner (Figure 1).

It is important to note that ghrelin’s activity is dependent on acylation by GOAT [3]. Consequently, expression of the *ghrelin* gene in a tissue is not automatically associated with expression of acylated ghrelin. So far, GOAT expression was demonstrated in human testis, ovary and placenta [22], indicating a local expression of acylated ghrelin and the importance of its activity in those tissues.

#### 4.1.2. Ghrelin’s Effect Reproductive Functions

Increased plasma acyl ghrelin levels correlate with conception success and endogenous desacyl ghrelin elevates pregnancy rate and number of pups at birth [105], indicating a positive effect of ghrelin on fertility. However, ghrelin, which can be found in embryos of different stages [16,35,36] with a decrease in concentration with cell division [35], has negative effects on embryo development, indicated by reduced blastocyst formation rates [165] as well as on embryo quality [161]. In contrast, ghrelin exerts a positive effect on implantation [185,187], supported by findings in animal models of ghrelin deficiency [187]. Moreover, ghrelin is not only detected in fetal tissue [38,39] but also binds to it [38], impacting fetus quantity negatively but fetus quality positively, indicated by a decreased ratio of number of fetuses per corpora lutea [179] and number of pups born per litter [189], but reduced pup mortality [155]. Besides ghrelin’s ability to stimulate fetal development resulting in increased birth weight [38], its significance in pregnancy is further supported by changes in circulating ghrelin. In humans, most data indicate elevated levels in the second trimester [77,78] with a decrease in ghrelin from second to third trimester [78,79], and thus low ghrelin levels during late pregnancy [77]. Moreover, ghrelin is also expressed in the placenta, but while in rats ghrelin is absent in the early part of pregnancy [40], in sheep and humans ghrelin mRNA was detected in the placenta throughout the whole pregnancy [40,43,44]. The placenta could be a source of fetal ghrelin, since ghrelin is more abundant in the umbilical vein than in the arteries [45]. Ghrelin is not only found to be expressed in mammary glands [62] but also in breast milk [63], with a decrease in total ghrelin concentration over time; thus, there were lower levels in the fifth compared to the second month after delivery [66]. Conversely, ghrelin stimulated cell proliferation of goat mammary epithelial cells [62] and increased milk yield [194]. In rats and humans, circulating ghrelin decreased during lactation [12,67,75] and normalized over time [73,85], which could be due to loss of ghrelin with breast milk. 

#### 4.1.3. Ghrelin’s Role in Reproduction-Related Health Conditions

Ghrelin may play a role in intrauterine growth. Although data describing the relation between ghrelin in the umbilical cord and maternal characteristics is not consistent [47,48,49], the majority of publications indicate a negative correlation between cord blood ghrelin and birth weight or length [45,46,50,53,54,55]. In contrast, there is a negative correlation between neonatal circulating ghrelin and birth weight or length [86,87,89]; thus, SGA, IUGR, and those weighing under 1500 g display increased circulating ghrelin levels compared to normal weight newborns [87,89]. Ghrelin may also be implicated in the pathogenesis of fertility and pregnancy-related health conditions. Firstly, patients suffering from PCOS display decreased circulating ghrelin levels compared to controls [201,202,203]. The extent of decrease of ghrelin in PCOS is supposedly depending on body weight and severity of PCOS [201,204], with an increase of ghrelin after treatment-induced normalization of hyperandrogenemia [201,205]. Secondly, observations that ghrelin was reduced in early onset preeclampsia, while it was increased in late onset preeclampsia [218], could explain why some studies report increased ghrelin in pregnancy-induced hypertension [213] and in mild and severe preeclampsia [216], while others describe reduced circulating ghrelin in preeclampsia [215,217]. Thirdly, a majority of studies in GDM observed decreased circulating ghrelin in patients compared to controls [64,65,88,219], with no difference 15 days after delivery in ghrelin serum between women with GDM and without diabetes [64], indicating a normalization with decreased disease activity. In addition, it should be kept in mind that inconsistencies in ghrelin levels in the circulation could be a result of variability in the methods of ghrelin measurement or lack of ghrelin stabilization in blood probes [84].

### 4.2. Nesfatin-1 

#### 4.2.1. Nesfatin’s Expression and Effects along the HPG Axis

**Figure 2:**
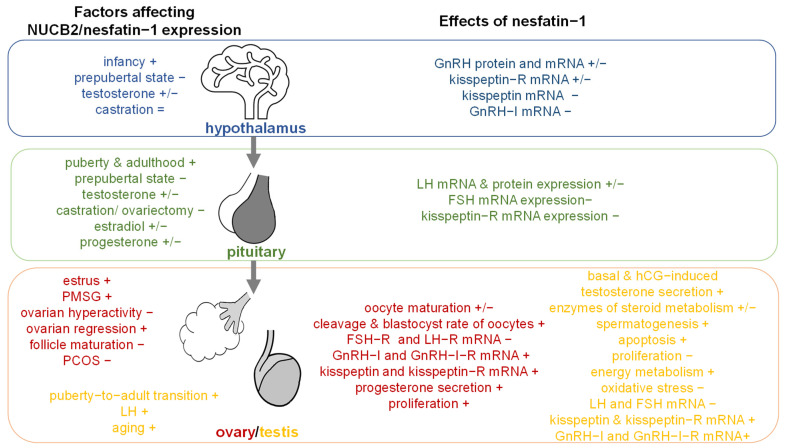
Nesfatin’s role along the HPG-axis. + increasing/stimulating or increased/stimulated; − reducing/ inhibiting or reduced/ inhibited; = not affecting/not changing or not affected/unchanged; R receptor; FSH follicle stimulating factor; LH luteinizing hormone; GnRH gonadotropin-releasing hormone; GnRH-I gonadotropin-releasing inhibiting hormone; PMSG pregnant mare serum gonadotropin; PCOS polycystic ovary syndrome.

NUCB2/nesfatin-1 is colocalized with GnRH in hypothalamic cells [224,225], with a trend of decreasing hypothalamic NUCB2 mRNA expression throughout neonatal to pre-pubertal development [227]. Moreover, testosterone decreases hypothalamic expression of NUCB2 mRNA [223]. In turn, nesfatin-1 reduces expression of hypothalamic genes for GnRH and kisspeptin [226], but does not affect circulating GnRH [252] (Figure 2).

NUCB2/nesfatin-1 is also expressed in [223,226,229] and binds to [228] pituitary tissue. Its expression is affected by sexual maturation, indicated by highest levels in pubertal rats and adult male rats [226], by testosterone, which increases NUCB2 mRNA and nesfatin-1 protein expression in the pituitary cells [223,224], as well as by estrogen that has different effects in vitro [224] than in vivo [9] on NUCB2 mRNA and nesfatin-1 protein expression. Nesfatin-1 reduces LHβ and FSHβ mRNA in the pituitary [226], also affecting circulating FSH and LH, but in dose- and application-dependent manner. Namely, nesfatin-1 is able to increase (icv, high dose) and decrease (ip, low dose) circulating FSH and LH [226,252] (Figure 2).

In various species NUCB2 mRNA is expressed in the testes [231,232,233], predominately in Leydig cells [226,232]. Noteworthy, NUCB2/nesfatin-1 expression in the testis is higher than in the hypothalamus and is increased by pituitary LH [232] and by aging [226,232]. Conversely, nesfatin-1 incubation of testis increases testicular testosterone secretion [226,231] by elevating expression of enzymes of steroid metabolism [226]. Additionally, in vivo peripheral testosterone is increased by high levels of icv applied nesfatin-1 [252]. In addition, nesfatin-1 incubation of testis stimulates spermatogenesis by promoting cell proliferation and survival as well as suppressing apoptosis and oxidative stress in testis [231], resulting in a positive effect of nesfatin-1 also on stress-induced testicular alterations [249,250,251] (Figure 2).

Moreover, NUCB2 mRNA and nesfatin-1 protein is expressed in the ovary [233,234] in a higher abundancy than in the hypothalamus [229]. Ovarian nesfatin-1 protein expression is affected by the estrus cycle with the highest concentration during estrus period [234], as well as by ovarian activity with low immunoreactivity in hyperactive and high signals in regressed ovaries [233]. On the cellular level, nesfatin-1-like immunoreactivity was detected in follicle cells [225], theca and granulosa cells [235], and interstitial cells of the ovary [229]. While in fish in vitro incubation with nesfatin-1 suppressed oocyte maturation [225,246], in porcine oocytes nesfatin-1 incubation stimulated cleavage and the blastocyst rate [235]. Intraperitoneally injected nesfatin-1 appears to have an inhibitory effect on ovarian expression, since it reduced ovarian mRNA expression of the LH and FSH receptors and increased expression of gonadotropin-inhibiting hormone and its receptor [225]. In granulosa cells, nesfatin-1 incubation increased progesterone secretion and cell proliferation [236], and in the circulation intraperitoneally injected nesfatin-1 decreases estradiol [246,247] (Figure 2).

#### 4.2.2. Nesfatin’s Expression in Other Reproductive Organs

NUCB2 mRNA and NUCB2/nesfatin-1 protein are expressed in uterus [234], in a higher density than in the hypothalamus [229], in epithelial cells of the endometrium and uterine glands [229]. It is affected by estrous cycle with highest expression during estrus [234] and stimulated by estradiol administration [234]. NUCB2/nesfatin-1 is also detected in the amnion and decidua of the placenta [240]. In more detail, in humans it is found in the syncytiotrophoblast throughout all trimesters [241], as well as in umbilical cord blood [242] and in breast milk [65]. Throughout pregnancy, NUCB2 mRNA and NUCB2/nesfatin-1 protein is detected in maternal circulation and decreases from gestational days 12 to 16 and 21 [240].

#### 4.2.3. Nesfatin’s Role in Reproduction-Related Health Conditions

Nesfatin-1 may play a role in fertility and pregnancy-related disorders, but current data is inconsistent. Regarding PCOS, some studies found increased circulating levels of nesfatin-1 peptide in patients suffering from PCOS compared to controls [245,253], while in others decreased circulating nesfatin-1 protein were shown in patients with PCOS [254,255]. Similarly, results from examinations of HG are contradicting; however, careful matching showed that nesfatin-1 peptide blood concentration increased in patients with HG compared to controls [211]. In preeclampsia, there is a report of decreased circulating NUCB2/nesfatin-1 protein levels [256], as well as of increased nesfatin-1 peptide concentration in circulation [217]. In subjects with GDM the data is clearer, since the majority of studies observed reduced circulating nesfatin-1 protein in patients with GDM [65,242,257,258,259]. Similarly, the association between nesfatin-1 peptide umbilical cord blood levels and neonatal weight was reproduced in different investigations, showing a negative correlation between nesfatin-1 protein and fetal birth weight [261,262,263].

## 5. Conclusions

This review demonstrates that, while there are many data about ghrelin’s implication in the HPG axis and reproduction-related functions and diseases, investigations about nesfatin’s importance in reproduction are rare. However, since the expression of nesfatin-1 in the testis, ovaries, and uterus were higher than in the hypothalamus [229], effects of nesfatin-1 on reproductive hormones in the pituitary are significant [226,252] and nesfatin-1 has positive effects on testicular functions [249,250,251]. Nesfatin-1 appears to have a crucial role in the HPG axis, which should be further investigated in the future. In addition, in future studies ghrelin’s significance in reproductive organs and functions should also be examined in more detail, since numerous findings in the studies presented in the current review were contradicting and inconsistent. The endogenous role of ghrelin and nesfatin-1 in reproduction especially need to be specified, for example by the use of knock-out models or protocols including acute inhibition of endogenous ghrelin or nesfation-1.
